# A Prognostic Survival Model of Pancreatic Adenocarcinoma Based on Metabolism-Related Gene Expression

**DOI:** 10.3389/fgene.2022.804190

**Published:** 2022-05-18

**Authors:** Lin-ying Xie, Han-ying Huang, Tian Fang, Jia-ying Liang, Yu-lei Hao, Xue-jiao Zhang, Yi-xin Xie, Chang Wang, Ye-hui Tan, Lei Zeng

**Affiliations:** ^1^ Bethune Institute of Epigenetic Medicine, The First Hospital of Jilin University, Changchun, China; ^2^ State Key Laboratory of Oncology in South China, Collaborative Innovation Center for Cancer Medicine, Sun Yat-sen University Cancer Center, Guangzhou, China; ^3^ Cancer Center, The First Hospital of Jilin University, Changchun, China; ^4^ Department of Neurology and Neuroscience Center, The First Hospital of Jilin University, Changchun, China; ^5^ Department of Endocrinology, China-Japan Union Hospital of Jilin University, Changchun, China; ^6^ Department of Hepatobiliary and Pancreatic Surgery, The First Hospital of Jilin University, Changchun, China

**Keywords:** pancreatic adenocarcinoma, metabolism, gene expression, prognostic, survival model

## Abstract

Accurately predicting the survival prospects of patients suffering from pancreatic adenocarcinoma (PAAD) is challenging. In this study, we analyzed RNA matrices of 182 subjects with PAAD based on public datasets obtained from The Cancer Genome Atlas (TCGA) as training datasets and those of 63 subjects obtained from the Gene Expression Omnibus (GEO) database as the validation dataset. Genes regulating the metabolism of PAAD cells correlated with survival were identified. Furthermore, LASSO Cox regression analyses were conducted to identify six genes (*XDH*, *MBOAT2*, *PTGES*, *AK4*, *PAICS*, and *CKB*) to create a metabolic risk score. The proposed scoring framework attained the robust predictive performance, with 2-year survival areas under the curve (AUCs) of 0.61 in the training cohort and 0.66 in the validation cohort. Compared with the subjects in the low-risk cohort, subjects in the high-risk training cohort presented a worse survival outcome. The metabolic risk score increased the accuracy of survival prediction in patients suffering from PAAD.

## Introduction

The global adenocarcinoma statistics in 2020 based on the GLOBOCAN estimates taken from the International Agency for Research on Adenocarcinoma demonstrate that pancreatic adenocarcinoma (PAAD) has a high fatality rate (466,000 deaths in 496,000 cases) because of its poor prognosis. Moreover, it is the seventh leading cause of adenocarcinoma death in both sexes (Sung et al., 2021). Pancreatic ductal adenocarcinoma (PDAC) is a deadly disease with a 5-year survival rate of approximately 9% (Flowers et al., 2021). Somatic mutations are the most prevalent genetic alterations such as *KRAS*, *GNAS*, and tumor suppressor genes such as *CDKN2A*, *TP53*, and *SMAD4* (Singhi and Wood, 2021). Other genes associated with DNA repair also contribute to PDAC development, including *BRCA2*, *ATM*, *PALB2*, *FANCC*, and *FANCG*. In addition to genetic mutations, PDAC involves molecular abnormalities such as hyperactivated growth factor signaling, dysregulated gene expression (transcriptional or posttranscriptional), epigenetic changes, and abnormal posttranslational modifications (Vaziri-gohar et al., 2018).

In PAAD, metabolic reprogramming, including rewired glucose, lipid, and amino acid metabolism, and abnormal metabolism characteristics within the tumor microenvironment, contribute to tumor progression. These phenomena are related to drug resistance to chemotherapy, radiotherapy, and immunotherapy (Qin et al., 2020). Genetic alterations and the tumor microenvironment related to PDAC development participate in the metabolic rewiring process (Dasgupta et al., 2019; Xu et al., 2020). Glycolytic flux is the main carbon metabolism process in all cells. It does not only produce adenosine triphosphate (ATP) but also provides biomass for anabolic processes that support cell proliferation. Increased expression levels of glucose transporters and rate-limiting enzymes that regulate the rate of glycolytic flux are increased (Akakura et al., 2001; Mikuriya et al., 2007; Commisso et al., 2013; Guillaumond et al., 2013), in addition to the elevated levels of glycolysis and pentose phosphate pathways being the characteristic of early tumors (Vernucci et al., 2019). Consequently, glycolytic metabolites, including lactate, are elevated in pancreatic cancer cells (Mikuriya et al., 2007; Guillaumond et al., 2013; Shi et al., 2014). Targeting glucose metabolism can sensitize pancreatic cancer to MEK inhibition and underlines the potential of co-targeting glycolysis and MAPK as an alternative approach to treating *KRAS*-driven PDAC (Yan et al., 2021).

Increased secretion of the arginine metabolite inducible nitric oxide (NO) synthase (iNOS) and endothelial nitric oxide synthase (eNOS) has been detected in PDAC tissues compared with normal tissues (Vickers et al., 1999; Lim et al., 2008). In PDAC, high levels of iNOS are associated with the proliferation and invasiveness of tumor cells (Wang et al., 2016). The function of NO and related signaling pathways in the monitoring of pancreatic cancer development and progression has been reported (Fujita et al., 2014; Wang et al., 2016). The importance of dysregulated NO in cellular glutamine metabolism is increasingly recognized in PDAC patients, which is integral to the invasive property of cancer cells and can stimulate angiogenesis and regulate oxidative phosphorylation. Given that PDAC patients exhibit an increased dependence on glutamine metabolism, small molecular inhibitors targeting the initiating enzyme GLS1 in glutamine metabolism have been actively investigated (Altman et al., 2016). Previous studies have reported that targeting glutamine metabolism can increase the sensitivity of PAAD to gemcitabine and improve its curative effect (Chen R. et al., 2017). Recently, clinical studies evaluating the combination of small molecular inhibitors and chemotherapy or targeted therapy against various solid tumors have been conducted (NCT02861300, NCT03965845, NCT04250545, NCT02771626, NCT03944902, and NCT03875313). Moreover, the safety, tolerability, and efficacy of these methods have been evaluated (Xu et al., 2020).

Accurate risk stratification is important for therapeutic decision-making and survival prediction. However, a metabolic signature panel has not been explored to accurately stratify patients suffering from PAAD to predict their prognosis and treatment management.

In this study, a prognostic survival model based on metabolic genes was constructed according to the gene expression data obtained from TCGA dataset. The model was further validated using the GEO dataset to explore an efficient metabolic signature to more accurately manage the stratification of PAAD.

## Materials and Methods

### Data Collection

Normalized RNA sequencing (fragments per kilobase million, FPKM) and relevant clinical data (sex, age, histological grade, AJCC-TNM stage, survival time, and survival status) for TCGA-PAAD were obtained from TCGA (https://portal.gdc.cancer.gov/). A total of 182 mRNA samples (178 PAAD and 4 normal tissues) were analyzed. The microarray data of 63 PAAD samples in GSE57495 based on GPL15048 (Rosetta/Merck Human RSTA Custom Affymetrix 2.0 microarray HuRSTA 2a520709.CDF) (Affymetrix, Tampa, FL, United States) were obtained from the GEO database (http://www.ncbi.nlm.nih.gov/geo/). The expression profile data were log2-transformed. Furthermore, the detailed clinicopathologic data, including disease stage, survival time, and survival status, were used. The two datasets underwent a batch correction process via the “sva” R package so that they were comparable.

### Construction and Validation of a Metabolic Risk Score

Data obtained from TCGA dataset were used to construct the metabolic risk score model, which was used as the training dataset. A total of 940 candidate metabolism-related genes (MRGs) were extracted considering KEGG pathway genes, 872 of which were common in the training dataset and GSE57495. The “limma” R package was used to identify differentially expressed MRGs (DEMRGs) (*p* ≤ 0.05 indicated that genes exhibited at least 1.5-fold changes) compared with normal tissues. After removing seven cases without follow-up, 171 cases with tumor samples and relevant clinical data were included in the subsequent analysis. Univariable Cox regression analysis was applied to assess the correlation of DEMRGs with PAAD patients’ overall survival rate (OS, *p* ≤ 0.01). Subsequently, these genes were categorized as the prognostic DEMRGs (PDEMRGs). Then, the least absolute shrinkage and selection operator (LASSO) Cox regression analysis was conducted to determine the best weighting coefficient for prognosis-metabolic genes. After conducting a 1,000,000-fold cross-validation on the maximum-likelihood estimate of the penalty, the minimum criterion was determined by using the optimal penalty parameter λ. Finally, a metabolic model was established. The GSE57495 dataset was used as the validation cohort. The patients in each dataset were divided into high- and low-risk cohorts based on the median risk score of the training dataset. Univariable and multivariable Cox regression analyses were performed to evaluate the independent prognostic value with respect to the metabolic risk score. *p* ≤ 0.05 was considered statistically significant.

### Gene Set Enrichment and Molecular Functional Relevance Analyses

Kyoto Encyclopedia of Genes and Genomes (KEGG) pathways were used to assess the significance of the metabolic risk scores. The gene set enrichment analysis (GSEA; GSEA v4.1.0 software, http://software.broadinstitute.org/gsea/login.jsp) was performed to evaluate the enriched pathways in the high- and low-risk cohorts. The metabolic pathway-related gene sets of “c2.cp.kegg.v7.4.symbols” was the reference gene set used in GSEA to be compared against. Any pathway with *p* ≤ 0.05 and a false discovery rate *q* ≤ 0.25 was considered statistically significant.

Molecular and functional relevance analyses of the PDEMRGs were performed using Metascape (http://metascape.org). The search tool for the retrieval of interacting genes (STRING; https://cn.string-db.org) was used to analyze the protein–protein interaction network.

### Validation of the Identified Metabolism-Related Mutations Through Public Computational Tools

The PAAD mRNA levels reported in the gene expression profiling interactive database (GEPIA, http://gepia.cancer-pku.cn/) were used to verify the PDEMRG expressions adopted in the proposed model. GEPIA corroborated the differences in the gene expression between PAAD (*n* = 179) and normal pancreatic tissues (*n* = 171).

Tumor immune estimation resource version 2 (TIMER2.0; http://timer.cistrome.org), Gene-DE, and Gene-Surv modules were used to analyze the differential PDEMRG expressions between tumor and adjacent normal tissues and their relationship with pan-cancer outcomes. The correlation between PDEMRG expressions and immune infiltration concerning different immune cell types was also obtained from TIMER2.0 database.

### Statistical Analysis

R packages “survival” and “survminer” were used to divide the subjects into high- and low-risk cohorts with respect to the median risk score. The receiver operating characteristic (ROC) curves and area under the curve (AUC) value calculated using the “survivalROC” package of the Rstudio software were used to identify the metabolic risk score accuracy. The Kaplan–Meier curves with log-rank tests were used to compare the survival rates with each other. Univariable and multivariable Cox regression analyses were performed for the subsequent clinical analyses. The resulting data were presented using the “pheatmap” and “ggplot2” packages of Rstudio.

## Results

### Identification of DMREGs in Pancreatic Adenocarcinoma Patients

In the training dataset, 77 DEMRGs were identified compared with normal tissues, among which there were 43 upregulated genes and 34 downregulated genes in PAAD (Supplementary Figure S1).

**FIGURE 1:**
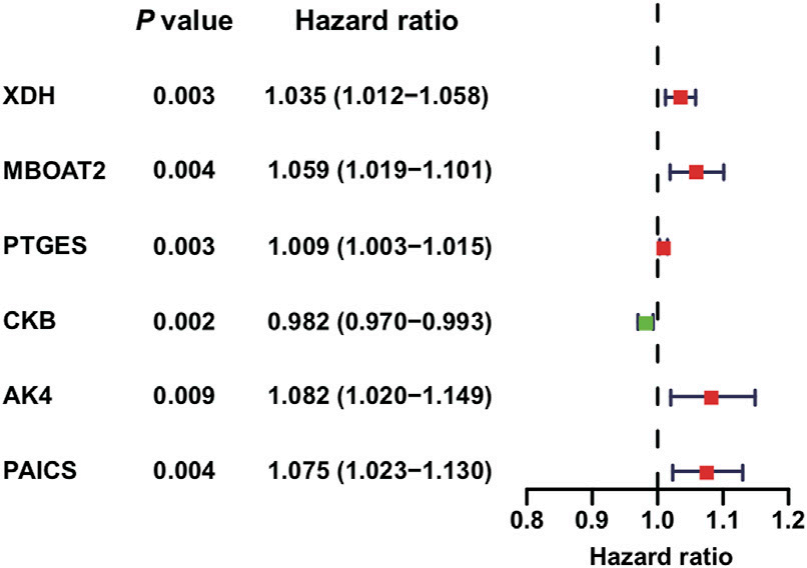
Identification of PDEMRGs in PAAD using TCGA database: forest plot of six PDEMRGs obtained from univariable Cox regression analysis (*p* ≤ 0.01): high-risk genes are represented in red (hazard ratios, HR > 1) and low-risk genes are represented in green (HR ≤ 1).

A total of six PDEMRGs (five high-risk genes and one low-risk gene) were significantly related to the OS in PAAD patients (Figure 1). The mRNA expressions of all six genes were upregulated in PAAD.

### Establishment and Validation of the Prognostic Risk Model

Using LASSO Cox regression analysis, six genes with high coefficients were selected to develop the metabolic risk score (Table 1). Among them, xanthine dehydrogenase (*XDH*), membrane-bound O-acyltransferase domain containing 2 (*MBOAT2*), prostaglandin E synthase (*PTGES*), adenylate kinase 4 (*AK4*), and phosphoribosylaminoimidazole carboxylase and synthase (*PAICS*) were identified as high-risk genes; while creatine kinase B-type (*CKB*) was identified as a low-risk gene. The formula for the metabolic risk score is as follows: metabolic risk score = (0.0208 × expression of *XDH*) + (0.0286 × expression of *MBOAT2*) + (0.0025 × expression of *PTGES*) + (0.0620 × expression of *AK4*) + (0.0229 × expression of *PAICS*) − (0.0140 × expression of *CKB*). The metabolic risk score of each subject in the training and validation cohorts was calculated according to this formula. Then, the subjects were divided into high- and low-risk groups based on the median score of the training cohort.

**TABLE 1:** Metabolic risk score with respect to the six genes developed according to LASSO Cox regression analysis.

Gene	Coef	Metabolic-related KEGG pathways
XDH	0.0208	Purine metabolism
MBOAT2	0.0440	Glycerolipid metabolism
PTGES	0.0025	Arachidonic acid metabolism
AK4	0.0062	Purine metabolism and thiamine metabolism
PAICS	0.0229	Purine metabolism
CKB	−0.014	Arginine and proline metabolism

**FIGURE 2:**
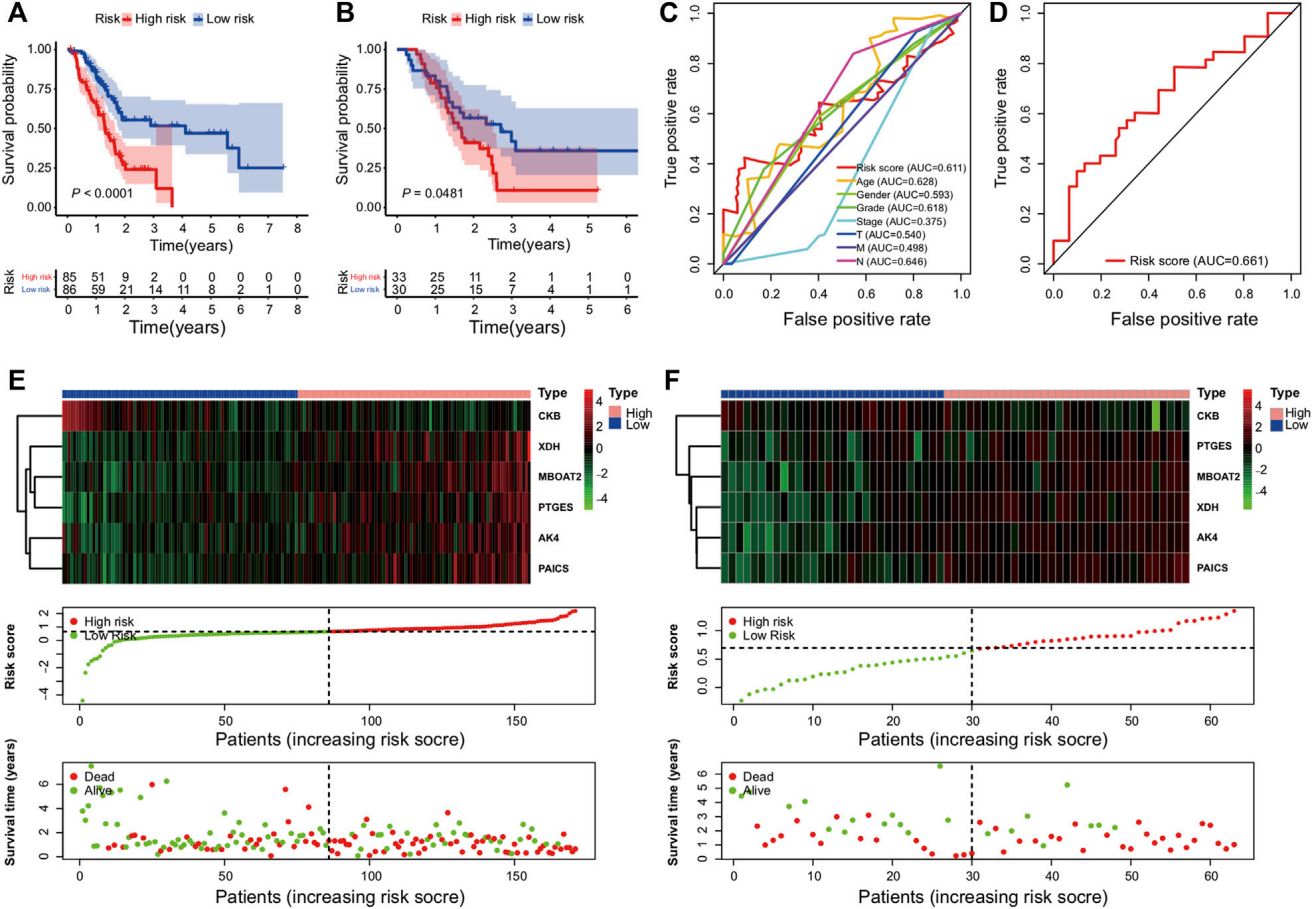
Establishment and validation of the metabolic genes prognostic risk model: **(A,B)** Kaplan–Meier curve analysis comparing the OS between patients in high-risk and low-risk groups in training and validation cohorts; **(C,D)** survival prediction ROC curves of the risk model and other clinical indices from two cohorts; **(E,F)** from top to bottom, there are the six PDEMRG expression patterns, risk score distribution of patients, and their survival status scatter plots in the training and validation cohorts.

The overall survival results of the patients in the high- and low-risk cohorts were compared using the Kaplan–Meier curves in the training (Figure 2A) and validation (Figure 2B) cohorts to identify the prognostic differences. The OS of the high-risk group was poorer than that of the low-risk group (*p* ≤ 0.05) (Figures 2A,B).

The ROC analysis was used to assess the sensitivity and specificity of the metabolic risk score. The 2-year survival AUCs were 0.61 and 0.66 in the training (Figure 2C) and validation cohorts (Figure 2D), respectively.

Heat maps were used to compare the expressions of six metabolic genes. In each dataset, their expressions slightly varied but overall remained relatively consistent (Figures 2E,F). Moreover, dot plots demonstrated that the survival rate of patients in the low-risk cohort was better than that of the patients in the high-risk cohort (Figures 2E,F).

### Univariable and Multivariable Analyses

**FIGURE 3:**
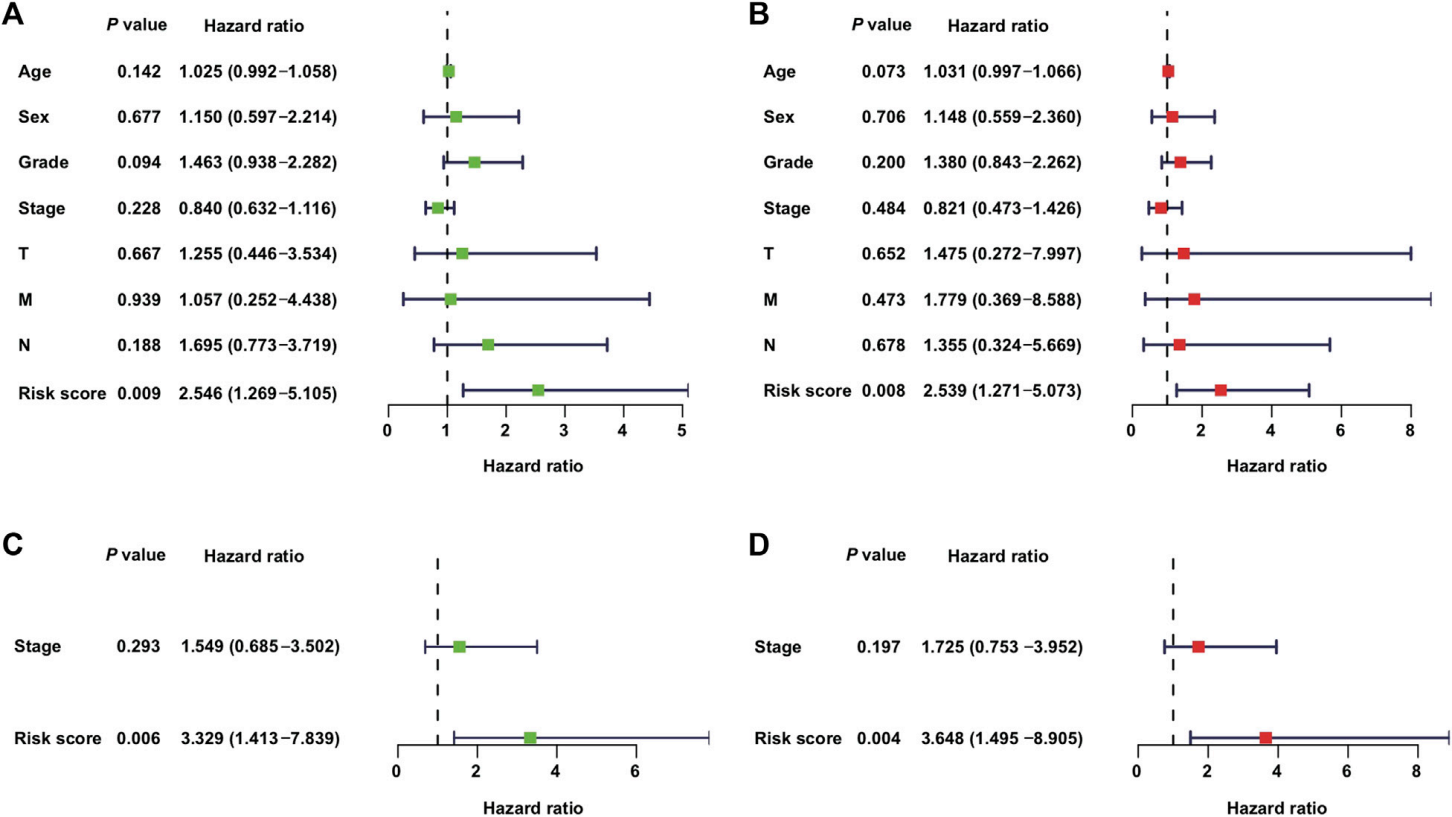
Independent value of the metabolism-related gene prognostic risk model: **(A,B)** forest plots of the univariable and multivariable Cox regression analyses of the relationship between risk model or clinical factors and OS in the training cohort; **(C,D)** forest plots of the univariable and multivariable Cox regression analyses in the validation cohort.

In addition to the metabolic risk score, the following values were examined in the training cohort: age, sex, grade, stage, T-stage, and N-stage (Figures 3A,B). Moreover, the stage values were assessed in the validation cohort regarding univariable and multivariable Cox regression analyses (Figures 3C,D).

The clinical covariates of the training and validation cohorts are listed in Table 2. Among other clinical factors, the results of multivariate analysis suggest that the metabolic risk is an independent prognostic factor, with hazard ratios of 2.539 (95% CI: 1.271–5.073) and 3.648 (95% CI: 1.495–8.905) in the training (Figure 3B) and validation cohorts (Figure 3D), respectively.

**TABLE 2:** Clinical covariates of the training and validation cohorts.

Characteristic	Training cohort (*n* = 175)	Validation cohort GSE (*n* = 63)
Sex		
Female	74 (42%)	—
Male	88 (51%)	—
Unknown	13 (7%)	
Age (years)		
≤60	52 (30%)	—
>60	110 (63%)	—
Unknown	13 (7%)	
Grade		
High	25 (14%)	—
Moderate	88 (51%)	—
Poor	49 (28%)	—
Unknown	13 (7%)	
Stage		
I	16 (9%)	13 (21%)
II	139 (80%)	50 (79%)
III	3 (2%)	—
IV	4 (2%)	—
Unknown	13 (7%)	
T-stage		
1	5 (3%)	—
2	20 (11%)	—
3	134 (77%)	—
4	3 (2%)	—
Unknown	13 (7%)	
N-stage		
0	45 (26%)	—
1	117 (67%)	—
Unknown	13 (7%)	
Metabolic risk score		
High	85 (50%)	33 (52%)
Low	86 (50%)	30 (48%)
Survival		
Alive	84 (48%)	21 (33%)

### Gene Set Enrichment and Molecular Functional Relevance Analyses

**FIGURE 4:**
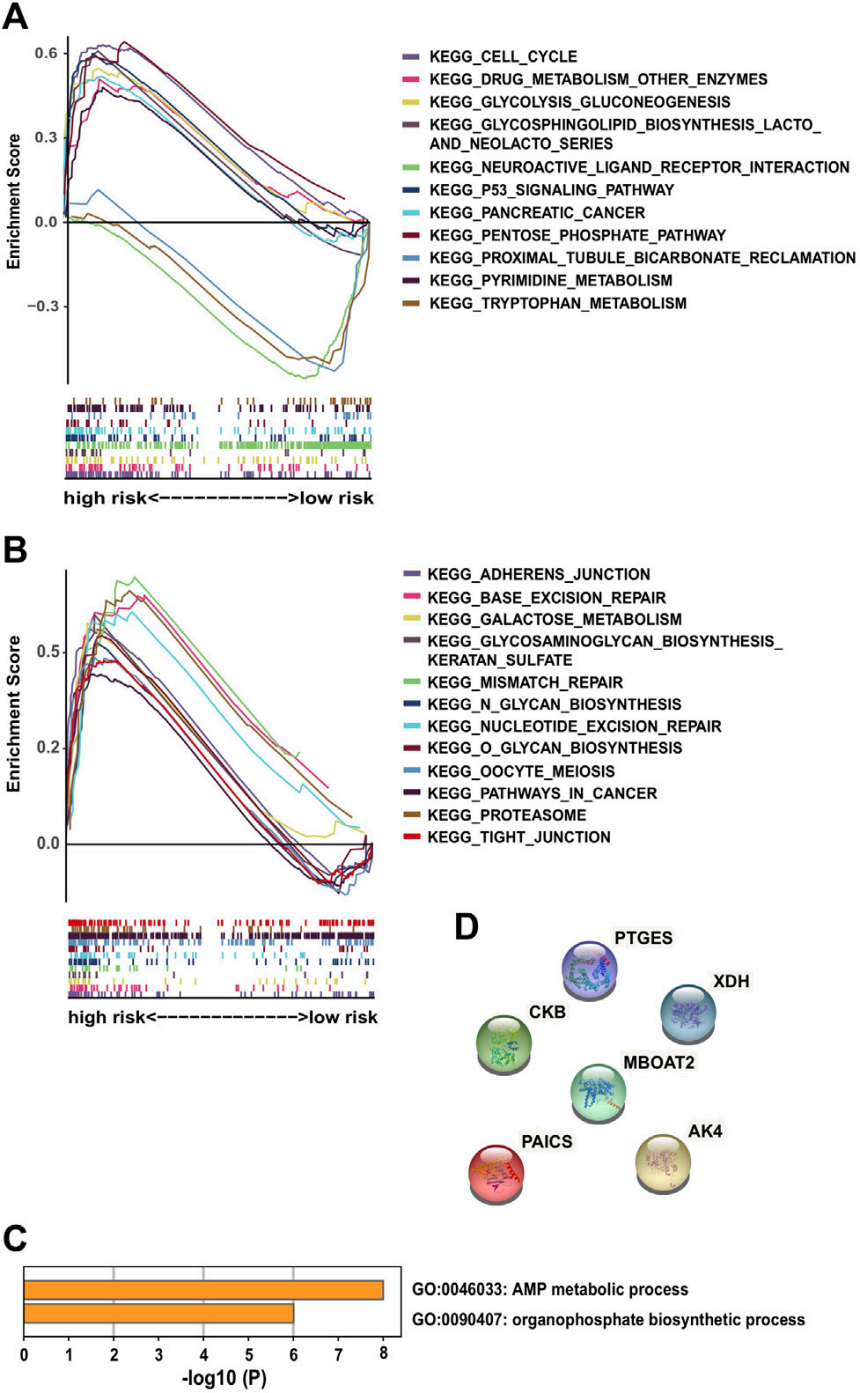
Significantly enriched KEGG pathways in the training cohort *via* GSEA and molecular functional relevance analysis: **(A,B)** enriched pathways of the high-risk group in the training cohort. **(C)** Enriched terms of GO-BP among the six PDEMRGs obtained from Metascape. **(D)** Protein–protein interactions among the six PDEMRGs obtained from STRING.

GSEA was performed on each dataset to explore metabolism-related and other enriched KEGG pathways associated with metabolic covariates. In the training cohort, a high-risk group with significant enrichment pathways was concentrated on the p53 signaling pathway, cell cycle, glycosphingolipid biosynthesis lacto and neolacto series, pentose phosphate, glycolysis gluconeogenesis, drug metabolism enzymes, pyrimidine metabolism, and pancreatic cancer pathways (Figure 4A).

Other identified pathways included the galactose metabolism glycosaminoglycan biosynthesis of keratan sulfate, adherens junction, tight junction, mismatch repair, base excision repair, nucleotide excision repair, and proteasome pathways. Moreover, the following pathways were present in cancer cells: O glycan biosynthesis, N glycan biosynthesis, and oocyte meiosis pathways (Figure 4B). The pathways with significant enrichment in the high-risk validation cohort included glycosaminoglycan degradation, DNA replication, and drug metabolism enzymes pathways (Supplementary Figure S2).

From the Metascape analysis results, two enriched gene ontology biological process terms (GO-BP) were obtained among the six PDEMRGs: the AMP metabolic (three genes) and organophosphate biosynthetic process results (four genes) (Figure 4C). The protein–protein interactions among the six PDEMRGs obtained from the STRING database were also analyzed, and no interactions were found (Figure 4D).

### Validation of the Identified Metabolism-Related Mutations Through Public Computational Tools

**FIGURE 5:**
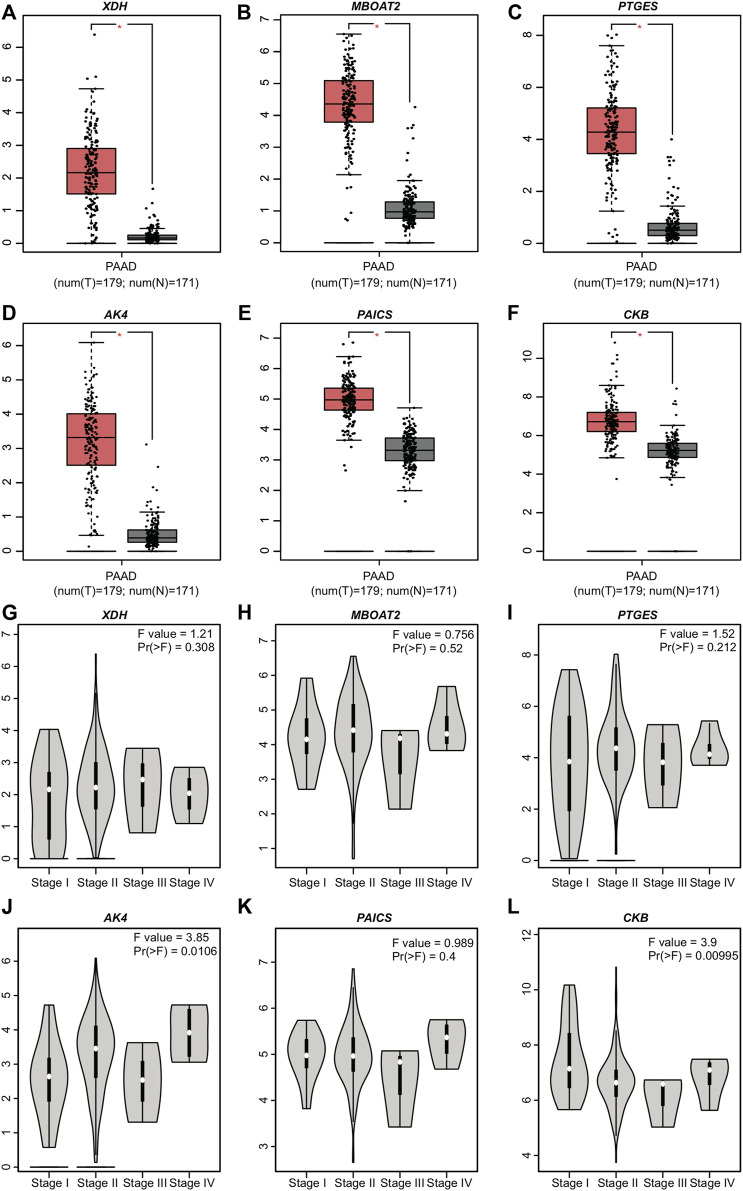
Expression levels of the six PDEMRGs obtained from GEPIA database: **(A–F)** mRNA expression levels of the six PDEMRGs in PAAD and normal pancreatic tissues obtained from GEPIA database (*p ≤ 0.05)—red represents PAAD and gray represents normal pancreatic tissues, and **(G–L)** mRNA expression levels of the six PDEMRGs in PAAD with disease stage obtained from GEPIA database.

GEPIA showed that all the genes included (*XDH*, *MBOAT2*, *PTGES*, *AK4*, *PAICS*, and *CKB*) in the model exhibited different PAAD mRNA expressions compared with normal pancreatic tissues (*p* ≤ 0.05) (Figure 5A). However, their expression levels did not differ with the stages (Figure 5B). *XDH*, *MBOAT2*, *PTGES*, *AK4*, *PAICS*, and *CKB* were upregulated and their results were consistent with those of the proposed model.

The correlations between the gene expression level in pan-cancer and other TCGA tumors, as well as the clinical outcomes, were analyzed using the TIMER database. The expressions of six PDEMRGs varied among TCGA tumors, which could be a prognostic factor in some tumors (Supplementary Figures S3–S5). The expression of *XDH* was higher in bladder urothelial carcinoma, cervical squamous cell carcinoma, endocervical adenocarcinoma, esophageal carcinoma, kidney renal clear cell carcinoma, kidney renal papillary cell carcinoma, lung adenocarcinoma, lung squamous cell carcinoma, and uterine corpus endometrial carcinoma, compared with corresponding normal tissues. However, its expression was lower in breast invasive carcinoma, cholangiocarcinoma, colon adenocarcinoma, liver hepatocellular carcinoma, and rectum adenocarcinoma. The *XDH* expression was a poor prognostic factor in adrenocortical carcinoma and kidney chromophobe but a good prognostic factor in liver hepatocellular carcinoma clinical outcomes (Supplementary Figures S3, S5). The correlation between the other five PDEMRG expression levels in pan-cancer and other TCGA tumors and clinical outcomes is documented in (Supplementary Figures S3–S5).

**FIGURE 6:**
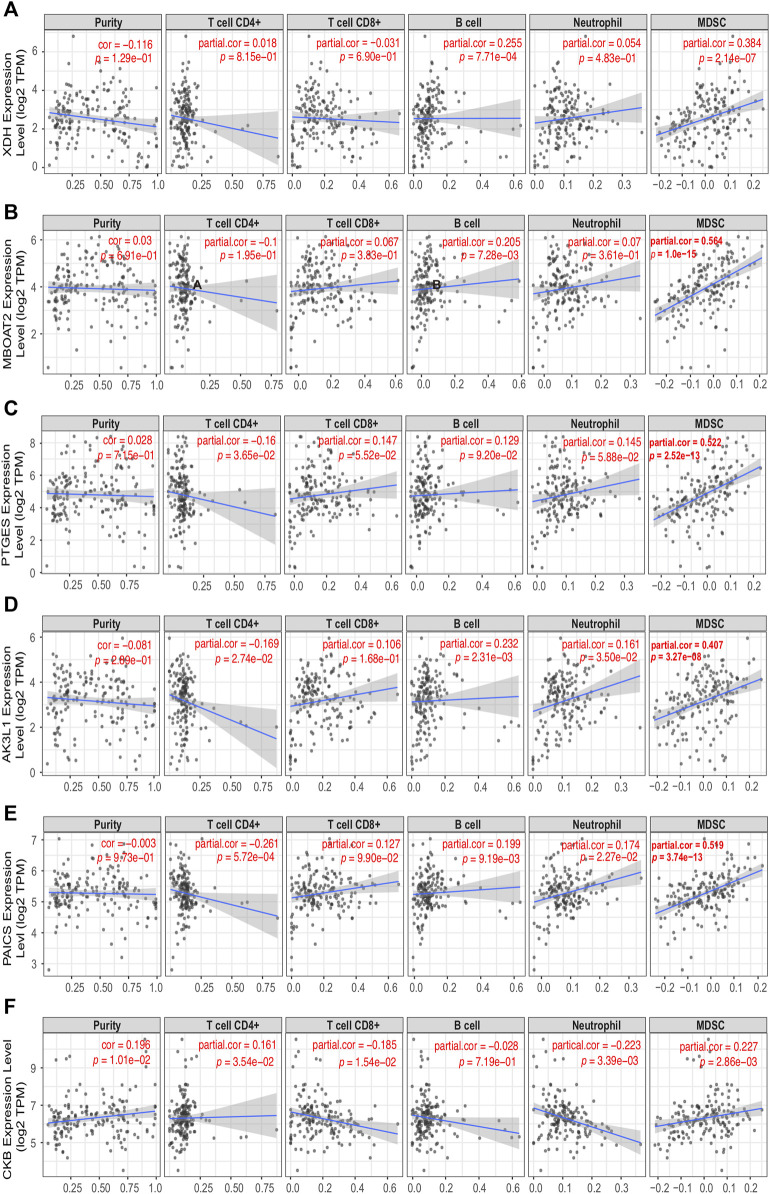
Correlation among the expressions of six PDEMRGs and different immune cell types in PAAD patients obtained using TIMER 2.0 database **(A–F)** Correlation among the expression levels of *XDH*, *MBOAT2*, *PTGES*, *AK3L1*, *PAICS*, and *CKB* and infiltration of immune cells in PAAD.

The correlations among the expression of six PDEMRGs and different types of immune cells in PAAD were also identified; they were correlated with the infiltration of tumor purity, CD4^+^ T cells, CD8^+^ T cells, B cells, neutrophils, and myeloid-derived suppressor cells (MDSC) (Figures 6A–F). The *CKB* expression was positively correlated with tumor purity (Cox = 0.196, *p* = 1.01e−02) and infiltration of some immune cells, including CD4^+^ T cells (Cox = 0.161, *p* = 3.54e−02), MDSC (Cox = 0.227, *p* = 2.86e−3). On the other hand, it was negatively correlated with CD8^+^ T cells (Cox = −0.185, *p* = 1.54e−02) and neutrophils (Cox = −0.223, *p* = 3.39e−3) (Figure 6F). The expression of *XDH* and *MBOAT2* was positively correlated with the infiltration of B cells (Cox = 0.255, *p* = 7.71e−04; Cox = 0.205, *p* = 7.28e−03) and MDSC (Cox = 0.384, *p* = 2.14e−7; Cox = 0.564, *p* = 1.0e−15) (Figures 6A,B). A negative correlation between *PTGES* expression and infiltration of CD4^+^ T cells (Cox = −0.16, *p* = 3.65e−02), and positive correlation with the infiltration of MDSC (Cox = 0.522, *p* = 2.52e−13) was observed (Figure 6C). The expressions of *AK3L1* and *PAICS* were positively correlated with the infiltration of B cells (Cox = 0.199, *p* = 9.19e−03; Cox = 0.232, *p* = 2.31e−03), neutrophils (Cox = 0.174, *p* = 2.27e−02; Cox = 0.161, *p* = 3.50e−02), and MDSC (Cox = 0.407, *p* = 3.27e−08; Cox = 0.519, *p* = 3.74e−13). On the other hand, they were negatively correlated with the infiltration of CD4+ T cells (Cox = −0.169, *p* = 2.74e−02; Cox = −0.261, *p* = 5.72e−04) (Figures 6D,E).

## Discussion

A novel six-metabolism-related gene prognostic risk score was constructed based on TCGA-PAAD dataset, whose results were validated using the GSE57495 dataset. GEPIA corroborated the differences in six-metabolism-related gene expression between PAAD tissues (*n* = 179) and normal pancreatic tissues (*n* = 171). Multivariable analysis demonstrated that the metabolic risk score was an independent prognostic factor in PAAD.

The formula of the metabolic risk score proposed in this study underlined that *CKB* was related to a favorable survival outcome; moreover, the other five genes (*XDH*, *MBOAT2*, *PTGES*, *AK4*, and *PAICS*) were associated with unfavorable survival outcomes.

*XDH* belongs to a group of molybdenum-containing hydroxylases that are involved in the oxidative metabolism of purines. These encoded proteins perform different mechanistic functions. *XDH* can be converted into xanthine oxidase through reversible sulfhydryl oxidation or irreversible proteolytic modification. *XDH* is highly expressed in the small intestine, duodenum, liver, and colon tissues. The expression of *XDH* decreased in some cancer types such as prostate, colon, breast, liver, bladder, and leukemia (Xu et al., 2019). Moreover, the low expression of *XDH* was associated with poor prognoses in various types of cancers, including colorectal cancer (Linder et al., 2009), early-stage gastric cancer (Linder et al., 2006), breast cancer (Linder et al., 2005), ovarian cancer (Linder et al., 2012), and hepatocellular carcinoma (Chen G.-L. et al., 2017; Sun et al., 2020; Lin et al., 2021). Indeed, decreased *XDH* may mediate immune evasion by affecting the immune cell infiltration into the tumor microenvironment (Lin et al., 2021). Other researchers have demonstrated that low *XDH* can induce cancer stem cell-related gene expression in hepatocellular carcinoma (Chen R. et al., 2017; Sun et al., 2020). Among non-small-cell lung cancer (NSCLC) patients who received adjuvant chemotherapy, high xanthine oxidase expression levels were associated with a better prognosis (Kim et al., 2011). Other researchers have reported that high tumoral *XDH* expression is an independent predictor of poor prognosis in patients with lung adenocarcinoma (Konno et al., 2012). Using the TIMER2.0, we found that the expression of *XDH* was a poor prognostic factor in adrenocortical carcinoma and kidney chromophobe but a good prognostic factor in liver hepatocellular carcinoma clinical outcomes. Furthermore, the expression of *XDH* varied among TCGA tumors (Supplementary Figures S3, S5). In our model, a high mRNA level of *XDH* could increase the risk score, resulting in poor survival in PAAD patients. The expression of *XDH* was positively correlated with the infiltration of B cells and MDSC in PAAD cases (Figure 6A).

*MBOAT2* is broadly expressed in the bone marrow, brain, esophagus, prostate, and skin. This gene is involved in phospholipid metabolism. The results of previous studies were consistent with the results of this study, indicating that *MBOAT2* was overexpressed in the neoplastic epithelia of pancreatic ductal adenocarcinoma and was inversely correlated with patient survival (Badea et al., 2008). The role of *circ-MBOAT2* in modulating tumor development and glutamine catabolism in pancreatic cancer has been confirmed in the literature (Zhou et al., 2021). *MBOAT2* is differentially expressed in various types of tumors (Supplementary Figure S3). The mRNA expression of *MBOAT2* might be responsible for the prognosis of multiple tumors. For example, the high mRNA level of *MBOAT2* can increase the risk of poor prognosis of adrenocortical carcinoma, bladder urothelial carcinoma, head and neck squamous cell carcinoma HPV+, liver hepatocellular carcinoma, mesothelioma, pheochromocytoma, paraganglioma, uterine corpus endometrial carcinoma, and uveal melanoma. However, it can decrease the risk of poor prognosis in breast invasive carcinoma-basal (Supplementary Figure S5). The expression of *MBOAT2* is positively correlated with the infiltration of B cells and MDSC in PAAD cases (Figure 6B).

The protein encoded by the *PTGES* gene is a glutathione-dependent prostaglandin E synthase. *PTGES* is biased-expressed in the placenta, urinary bladder, appendix, skin, and testis. *PTGES* can produce prostaglandin E2 (*PGE2*) through the pro-inflammatory cytokine interleukin 1 beta (*IL1B*). In addition, *PGE2* mediates inflammation, pain, and fever (Ackerman et al., 2008; Wang et al., 2019). *PTGES* can promote bone cancer growth and bone cancer pain in mice (Isono et al., 2011). In patients with NSCLC, the expression of *PTGES* is significantly elevated and strongly related to poor clinical outcomes (Wang et al., 2019). *PTGES/PGE2*-signaling promotes lung metastasis in a lung tumor suppressor gene Gprc5a-knockout mouse model by creating an immunosuppressive microenvironment (Wang et al., 2020). The expression of *PTGES* varies among TCGA tumors and is related to the poor prognosis of glioblastoma multiforme, kidney renal clear cell carcinoma, liver hepatocellular carcinoma, rectal adenocarcinoma, and uveal melanoma. However, it is related to the good prognosis of head and neck squamous cell carcinoma HPV+ (Supplementary Figures S3, S5). The expression of *PTGES* is negatively correlated with the infiltration of CD4^+^ T cells but positively correlated with the infiltration of MDSC in PAAD cases (Figure 6C).

*AK4*, also known as “*AK3L1*,” is a member of the adenylate kinase enzyme family that is involved in energy metabolism. *AK4* is biased-expressed in the kidney, liver, fat, heart, skin, and brain tissues. The encoded protein is localized to the mitochondrial matrix and can regulate the adenine and guanine nucleotide compositions within a cell by catalyzing the reversible transfer of phosphate groups among these nucleotides. Subsequently, it affects stress, ATP regulation, drug resistance, hypoxia tolerance, and malignant transformation in cancer (Fujisawa et al., 2016). Previous studies have found that *AK4* is a poor prognosis marker of lung cancer (Jan et al., 2012) because it can negatively regulate the transcription factor *ATF3* to promote the metastasis of lung cancer (Jan et al., 2012; Kong et al., 2013). *AK4* also acts as a carcinogen in ovarian carcinoma (Tan et al., 2021) and is associated with multidrug resistance in osteosarcoma cell lines (Lei et al., 2018). The expression of *AK3L1* varies among TCGA tumors and is negatively correlated with the prognosis of cervical squamous cell carcinoma, endocervical adenocarcinoma, head and neck squamous cell carcinoma, head and neck squamous cell carcinoma HPV+, kidney chromophobe, liver hepatocellular carcinoma, lung adenocarcinoma, stomach adenocarcinoma, uterine corpus endometrial carcinoma, and uveal melanoma. However, it is positively correlated with the clinical outcomes of lymphoid neoplasm diffuse large B-cell lymphoma (Supplementary Figures S4, S5). The expression of *AK3L1* is positively correlated with the infiltration of B cells, neutrophils, and MDSC but negatively correlated with the infiltration of CD4+ T cells in PAAD cases (Figure 6D).

*PAICS* encodes a bifunctional enzyme that catalyzes purine biosynthesis and contains phosphoribosylaminoimidazole carboxylase activity in its N-terminal region and phosphoribosylaminoimidazole succinocarboxamide synthetase in its C-terminal region. *PAICS* is ubiquitously expressed in the placenta, appendix, adrenal gland, lymph node, testis, and liver. *PAICS*, a *de novo* purine metabolic enzyme, is significantly overexpressed in several tumor types, including lung adenocarcinoma, breast cancer, diffuse large B-cell lymphoma, and prostate cancer (Chakravarthi et al., 2017; Akashi et al., 2019; Zhou et al., 2019). The expression of *PAICS* varies among TCGA tumors and is a poor prognosis factor responsible for breast invasive carcinoma, breast invasive carcinoma-LumA, cervical squamous cell carcinoma, endocervical adenocarcinoma, head and neck squamous cell carcinoma, kidney chromophobe, kidney renal papillary cell carcinoma, brain lower grade glioma, liver hepatocellular carcinoma, lung adenocarcinoma, mesothelioma, sarcoma, and thyroid carcinoma (Supplementary Figures S4, S5). The expression of *PAICS* is positively correlated with the infiltration of B cells, neutrophils, and MDSC but negatively correlated with the infiltration of CD4+ T cells in PAAD cases (Figure 6E).

*CKB* encodes a cytoplasmic enzyme that is a member of the ATP:guanido phosphotransferase protein family involved in energy homeostasis. It can reversibly catalyze the transfer of phosphate between ATP and various phosphagens such as creatine phosphate. It is broadly expressed in the colon, brain, prostate, and stomach tissues. The mRNA expression level of *CKB* increases with an unmethylated *CKB* promoter in hematologic malignancies (Ishikawa et al., 2005). However, public RNA-seq datasets indicate that *CKB* is downregulated in human solid tumors, and its lower expression is associated with a worse prognosis in cervical, head–neck, colon (Mooney et al., 2011), gastric (Mello et al., 2015), kidney, ovarian, pancreatic and sarcoma prostate cancer patients (Wang et al., 2021). Another study reported that the *CKB* expression level is increased in some ovarian cancer tissues, and the knockdown of *CKB* can delay disease progression by decreasing glycolysis (Li et al., 2013). The expression of *CKB* varies among TCGA tumors and is associated with a good prognosis of cervical squamous cell carcinoma, endocervical adenocarcinoma, and kidney chromophobe. However, it is associated with poor prognosis of thyroid carcinoma prognosis (Supplementary Figures S4, S5). We identified *CKB* as a low-risk gene. In addition, the public transcriptomic data reported in TIMER 2.0 identified a negative correlation between *CKB* and infiltration of immune cells in PAAD patients. This serves as another validation method for our analysis. Although we have identified *CKB* as a low-risk gene; public transcriptomic data reported in TIMER 2.0 identified a negative correlation between *CKB* and infiltration of CD8+ T cells and neutrophils in PAAD patients (Figure 6F).

GSEA demonstrated that the most-abundant metabolism-related pathways are concentrated in the high-score risk score groups. Regarding the enrichment of genes regulating the glycosphingolipid biosynthesis of lacto and neolacto series, pentose phosphate and glycolysis gluconeogenesis, galactose metabolism, glycolysis gluconeogenesis, pyrimidine metabolism, galactose metabolism, O glycan biosynthesis, N glycan biosynthesis, and glycosaminoglycan biosynthesis–keratan sulfate pathway, the related biosynthesis pathway indicates an increased nutrient demand by cancer cells. Other pathways, including the p53 signaling pathway, cell cycle, adherens junction, tight junction, pancreatic cancer, base excision repair, nucleotide excision repair, mismatch repair, and proteasome pathways, in cancer indicate the promotion of cell biosynthesis.

The most involved GO-BPs among the six PDEMRGs were the AMP metabolic process and organophosphate biosynthetic process. They might synergize to transduce a molecular pathway even though there were no protein–protein interactions described between them until now. Note that this needs to be further verified.

The expressions of six PDEMRGs in pan-cancer were obtained from TCGA, and their association with outcomes was obtained from TIMER2.0 database. Accordingly, we can observe that these genes are crucial in the development of different tumor types. The expressions of six PDEMRGs were associated with immune infiltration in PAAD patients.

This study had several important limitations. First, it was not possible to obtain more clinical information using the data obtained from the GEO database. Second, it was not possible to adjust the data regarding the impact of therapy on survival rates. Therefore, the score obtained should be considered as prognostic rather than predictive because therapeutic factors cannot be excluded. Third, in real-world applications, the significance of the metabolic-related gene risk model should be further confirmed. To that end, basic experiments should be conducted to explore the potential pathogenesis, which include, but are not limited to, the use of the six PDEMRGs siRNA/cDNA *in vitro* to transfect pancreatic cancer cell lines to silence or overexpress target genes or inhibit the expression or function of high-risk genes with inhibitors. Then, cell viability, cell cycle, apoptosis, tumor metastasis, and invasion should be evaluated, which require other phenotypic experiments. *In vivo* experiments include utilizing PDEMRG inhibitors on xenogeneic tumor transplanted mice and the transplantation of ordinary tumor cells and gene knockout/overexpression tumor cells to observe the tumor growth and metastasis ability. Further research should be conducted to study the signaling pathways affecting the influence of PDEMRGs on tumor growth and interaction with their corresponding signal pathway markers. With the wide application of second-generation sequencing technologies in clinical practice, researchers will be able to conduct prospective research using our model.

In conclusion, a prognostic survival model for PAAD cases based on the expressions of metabolism-related genes was developed and validated in this study. Multivariable analyses showed that the metabolic risk score was an independent predictor of the survival rate and reflected the disordered metabolism of PAAD patients.

This risk model can be used as an effective method to predict the prognosis of PAAD patients.

## Data Availability

The datasets presented in this study can be found in online repositories. The names of the repository/repositories and accession number(s) can be found below: The website of GEO dataset is https://www.ncbi.nlm.nih.gov/geo/query/acc.cgi?acc=GSE57495. The data from TCGA accession ID is TCGA-PAAD.

## Abbreviations

ATP, adenosine triphosphate; AUCs, areas under the curve; CI, confidence interval; DEGs, differentially expressed genes; DEMRGs, differentially expressed MRGs; eNOS, endothelial nitric oxide synthase; FPKM, fragments per kilobase million; GEPIA, Gene Expression Profiling Interactive Analysis database; GEO, Gene Expression Omnibus; GSEA, gene set enrichment analysis; HR, hazard ratio; iNOS, inducible nitric oxide synthase; KEGG, Kyoto Encyclopedia of Genes and Genomes; LASSO, least absolute shrinkage and selection operator; MDSC, myeloid-derived suppressor cells; MRGs, metabolism-related genes; NSCLC, non-small-cell lung cancer; PAAD, pancreatic adenocarcinoma; PDAC, pancreatic ductal adenocarcinoma; PGE, prostaglandin E2; OS, overall survival; STRING, Search Tool for the Retrieval of Interacting Genes; ROC, receiver operating characteristic; TCGA: the Adenocarcinoma Genome Atlas; TME, tumor microenvironment.
