# Supplementary figures and images for: A Prognostic Survival Model of Pancreatic Adenocarcinoma Based on Metabolism-Related Gene Expression
Source: Front Genet. 2022 May 18;13:804190. doi: 10.3389/fgene.2022.804190 (PMC9158121; doi:10.3389/fgene.2022.804190)

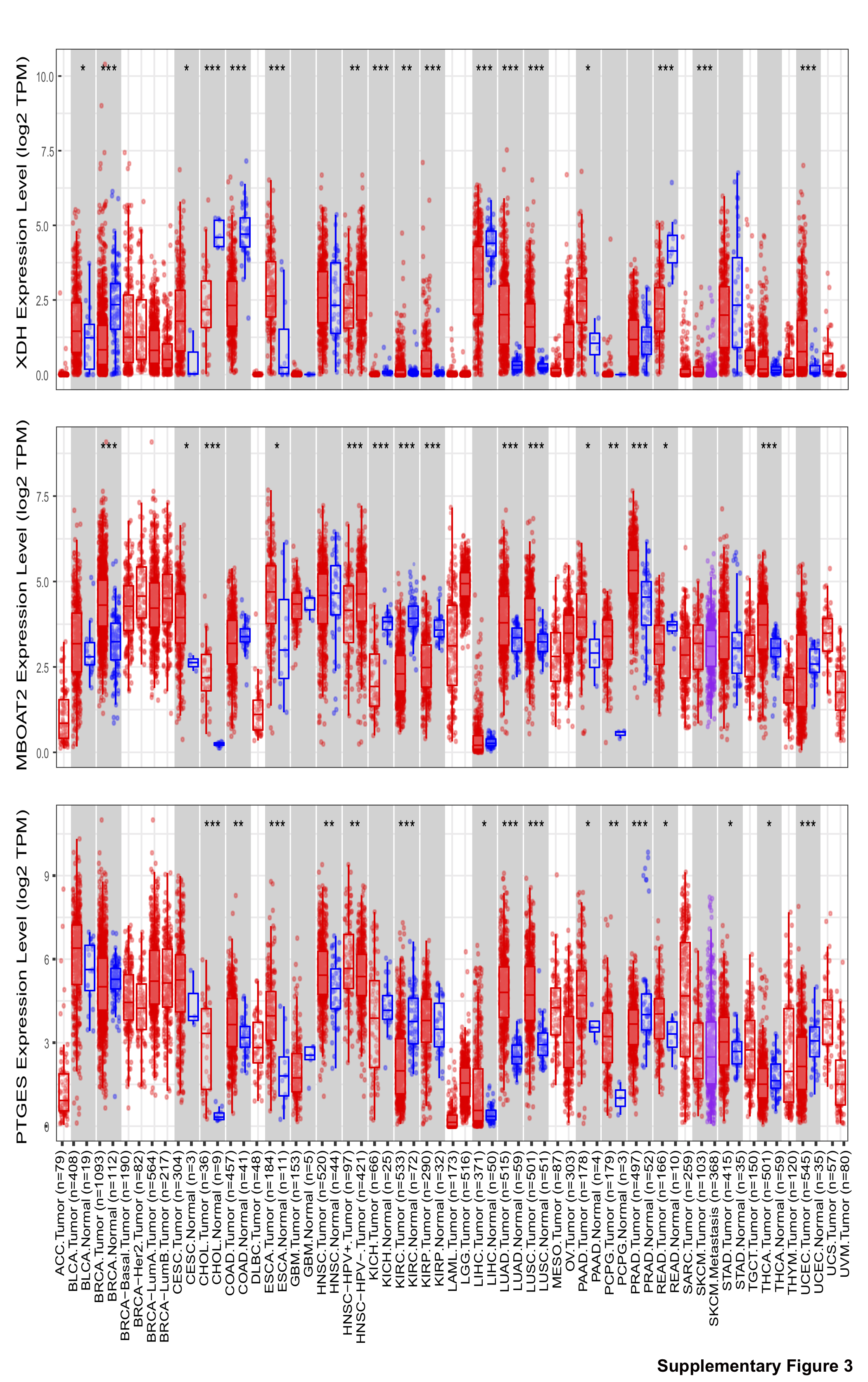

Supplement: Supplementary file 1 [file Image3.TIFF]

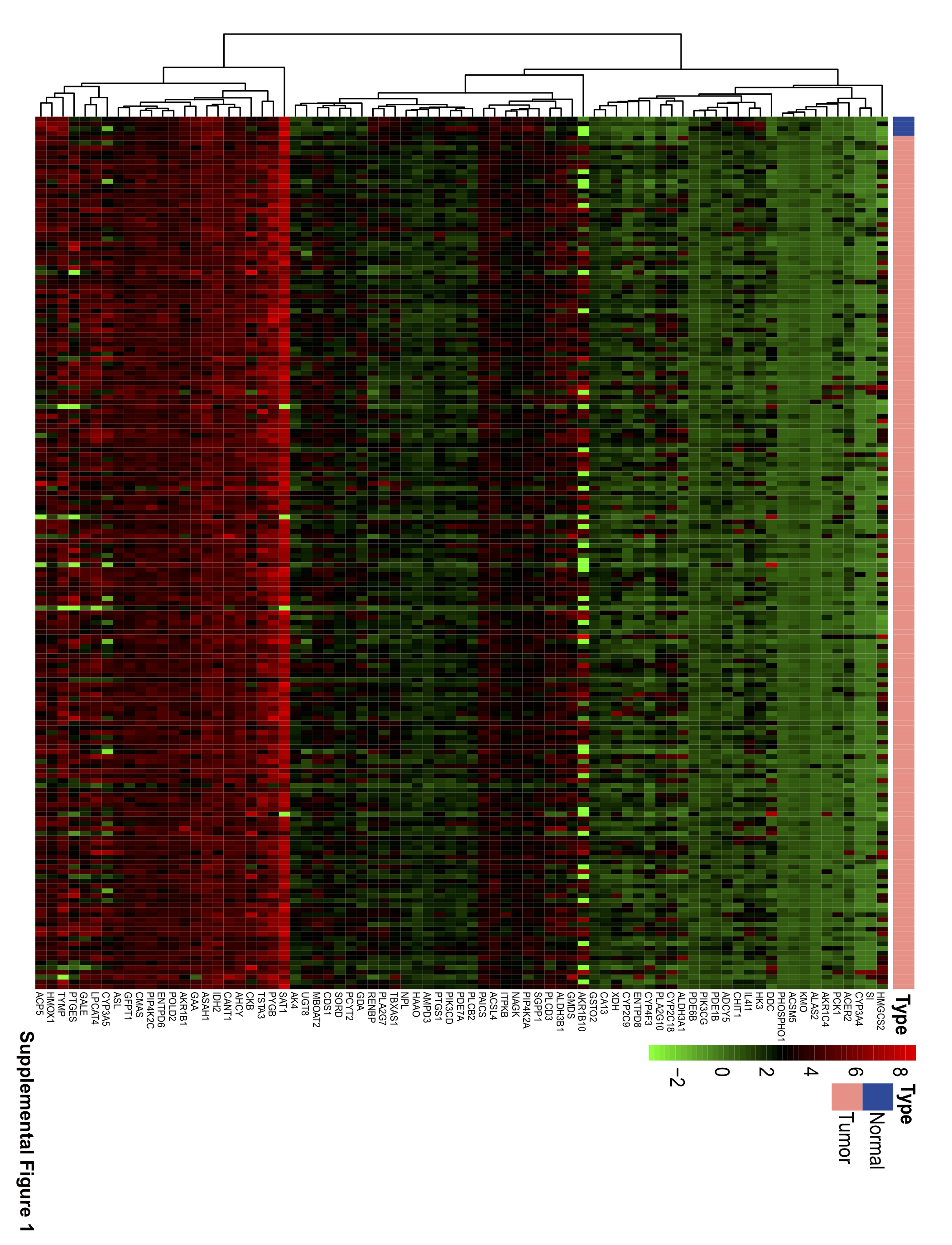

Supplement: Supplementary file 2 [file Image1.TIFF]

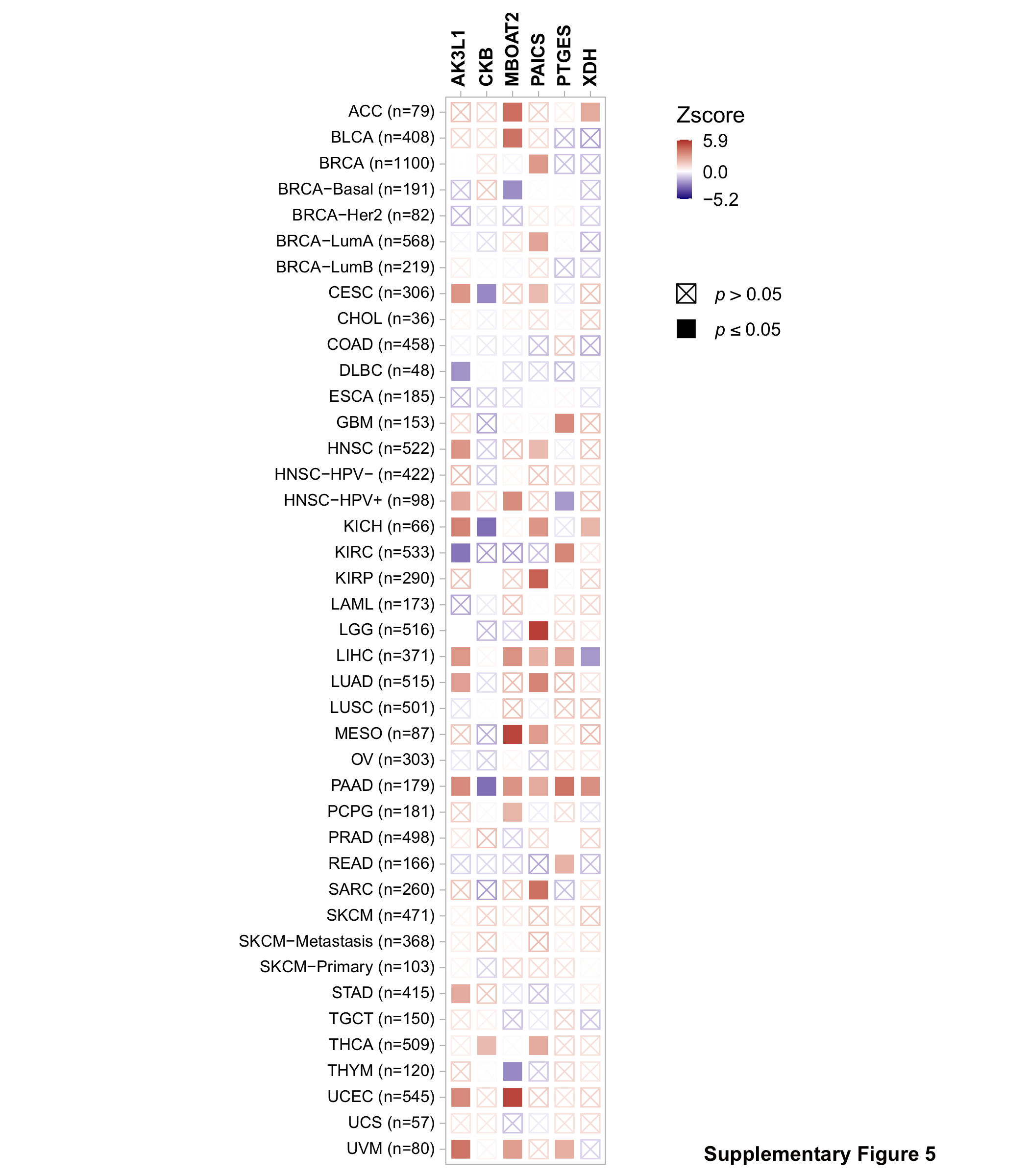

Supplement: Supplementary file 3 [file Image5.TIFF]

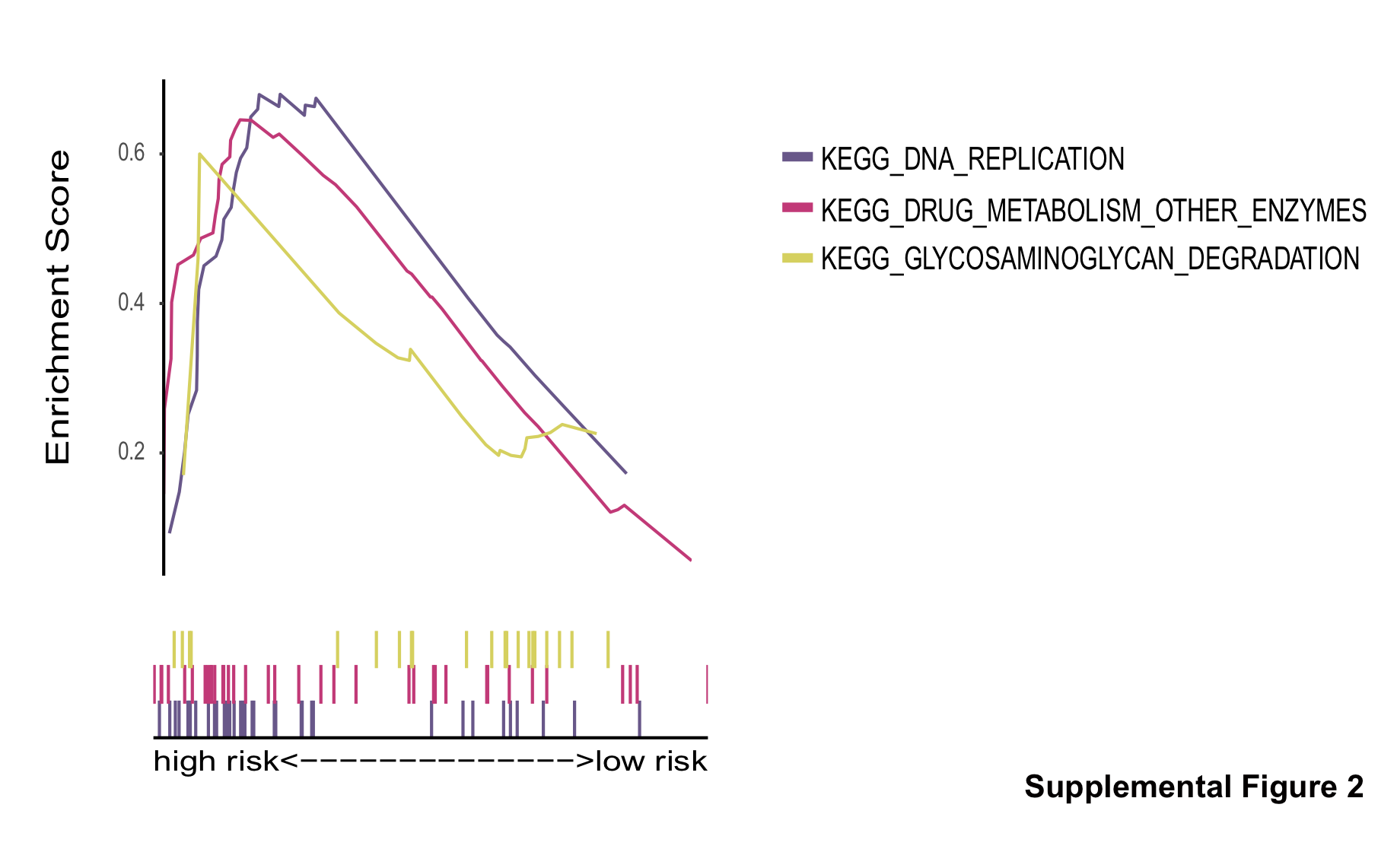

Supplement: Supplementary file 4 [file Image2.TIFF]

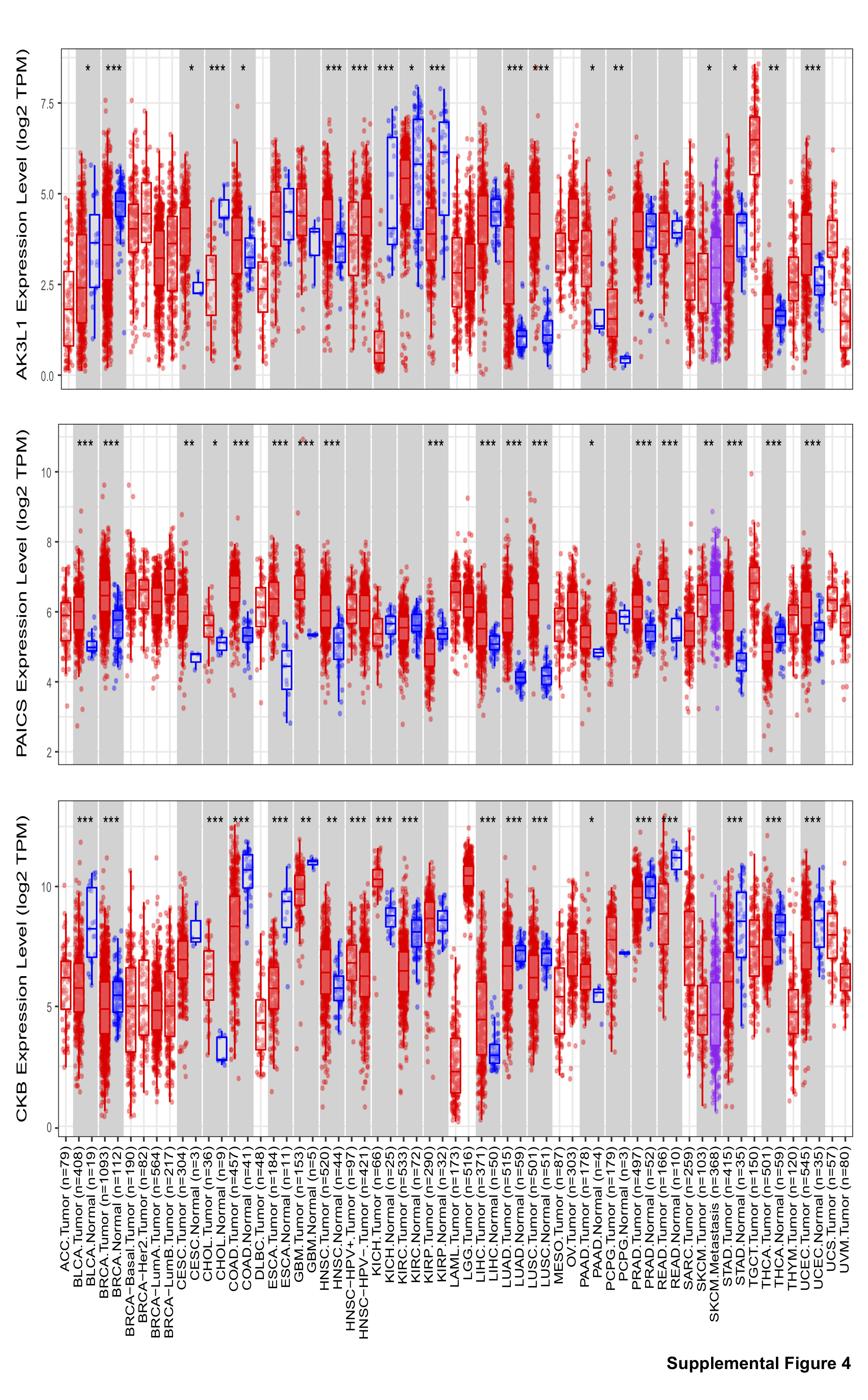

Supplement: Supplementary file 5 [file Image4.TIFF]
